# North Pacific warming shifts the juvenile range of a marine apex predator

**DOI:** 10.1038/s41598-021-82424-9

**Published:** 2021-02-09

**Authors:** Kisei R. Tanaka, Kyle S. Van Houtan, Eric Mailander, Beatriz S. Dias, Carol Galginaitis, John O’Sullivan, Christopher G. Lowe, Salvador J. Jorgensen

**Affiliations:** 1 Monterey Bay Aquarium, Monterey, CA 93940 USA; 2 Nicholas School of the Environment, Duke University, Durham, NC 27708 USA; 3 Department of Biological Sciences, California State University Long Beach, Long Beach, CA 90815 USA; 4 Present Address: Institute of Marine Sciences, University of California, Santa Cruz, CA 95064 USA; 5 Present Address: Pacific Islands Fisheries Science Center, National Oceanic and Atmospheric Administration, Honolulu, HI 96818 USA

**Keywords:** Biogeography, Climate-change impacts, Climate-change ecology

## Abstract

During the 2014–2016 North Pacific marine heatwave, unprecedented sightings of juvenile white sharks (*Carcharodon carcharias*) emerged in central California. These records contradicted the species established life history, where juveniles remain in warmer waters in the southern California Current. This spatial shift is significant as it creates potential conflicts with commercial fisheries, protected species conservation, and public safety concerns. Here, we integrate community science, photogrammetry, biologging, and mesoscale climate data to describe and explain this phenomenon. We find a dramatic increase in white sharks from 2014 to 2019 in Monterey Bay that was overwhelmingly comprised of juvenile sharks < 2.5 m in total body length. Next, we derived thermal preferences from 22 million tag measurements of 14 juvenile sharks and use this to map the cold limit of their range. Consistent with historical records, the position of this cold edge averaged 34° N from 1982 to 2013 but jumped to 38.5° during the 2014–2016 marine heat wave. In addition to a poleward shift, thermally suitable habitat for juvenile sharks declined 223.2 km^2^ year^−1^ from 1982 to 2019 and was lowest in 2015 at the peak of the heatwave. In addition to advancing the adaptive management of this apex marine predator, we discuss this opportunity to engage public on climate change through marine megafauna.

## Introduction

Marine ecosystems are exhibiting major ecological realignments in response to climate change^1,2^. Even though all species and taxa are influenced^3^, it has been argued that prioritizing attention to marine megafauna may help increase public concern and engagement to combat climate change^1,4^. However, the swift pace of changes in the ocean has driven a corresponding rapid shift in the distribution of marine megafauna^5,6^. As a result, a combination of conventional techniques as well as innovative and adaptive strategies for monitoring these species are needed. Such advances may also serve to help understand the broad and cascading consequences of our rapidly warming ocean to local ecosystems, communities and economies.

Except for rare instances^7^, adult white sharks (*Carcharodon carcharias*) are apex predators throughout their distribution^8^. Most juvenile sharks (< 2.5 m total body length: TBL)^9–11^ reside in coastal nursery areas^12^. In the northeastern Pacific this demographic consists of neonates (< 1.50 m TBL), young-of-year (“YOY”, < 1.75 m TBL), and other juveniles (< 2.5 m TBL) which primarily use coastal waters of northern Mexico and southern California^13,14^. A lack of confirmed observations beyond this region suggests the Southern tip of the Baja peninsula approximates their southernmost range. Within this region, juveniles remain in a relatively narrow temperature band, a pattern which is consistent across distinct white shark populations^15^. Even though white sharks are endothermic, greater surface-to-volume ratios in these juveniles may challenge their thermal inertia and ability to thermoregulate especially at the cold limit of their thermal habitat. As a result, the thermal range of juvenile white sharks is likely a major driver of the habitats they occupy, and this appears to be sensitive to climatic shifts during El Niño events^15^.

Ecosystems in the Northeast Pacific experienced extreme conditions over the past decade, highlighted by anomalously warm conditions from 2013 to 2016^16,17^. A warm mass of surface water described both as the Pacific Warm Anomaly and “the Blob” entered southern California in the fall of 2014^16^ and resulted in persistent warm-water conditions that endured to the 2015–2016 and 2018–2019 El Niño events^18^. Record-high sea surface temperature (SST) was observed in the area between Point Conception (34.4° N, 120.5° W) and San Miguel Island (34.04° N, 120.37° W), where nearshore SST reached a peak of 6.2 °C above the historical average in September 2015^19^. The multiyear warm anomalies caused widespread species shifts^20,21^, epizootics^22^, multiple unusual mortality events and dieoffs^23–26^, and broad impacts to commercial fisheries^27^ across the region.

**Figure 1:**
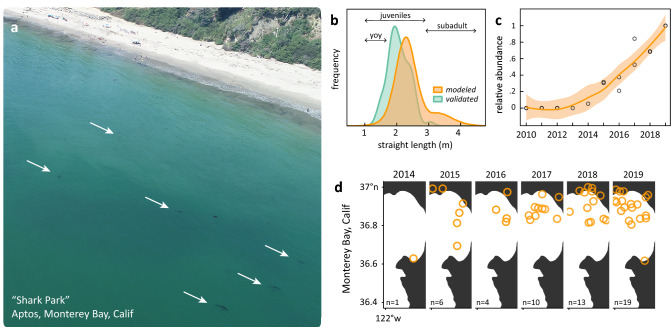
Juvenile white sharks became abundant in Monterey Bay during the 2014–2016 marine heatwave. **a** Aerial drone image detecting 6 small white sharks (denoted by white arrows) in September 2019 near Seacliff State Beach (36.97° N, 121.93° W). **b** Size composition from observer logbooks (n = 403) and photogrammetry validated measurements (*n* = 65) indicate the vast majority of sharks in this area are juveniles, with fewer subadults, and a small minority of young-of-the-year (“YOY”). **c**, Ensemble time series of Juvenile and YOY white shark abundance in Monterey Bay, from observer logbooks and a community science project with iNaturalist. A smoothing spline (solid line, shaded area is SE) summarizes the trend of each rescaled series, showing a constant increase in abundance from 0 juvenile and YOY sharks before 2014 to a peak in 2019. **d** Mapped iNaturalist positions of juvenile and YOY white sharks show a concentration in northern Monterey Bay near Aptos.

Concurrent with these ecologically significant warm-water events, local communities around the Monterey Bay (36.8° N, 121.95° W) began reporting small white sharks < 2.5 m TBL in nearshore Monterey Bay proximate human activities (Fig. 1a). Though juvenile white sharks 2.5–3.0 m TBL are detected north of Point Conception^13,14,28^, neonate, YOY, and other juveniles < 2.5 m TBL sharks in the Monterey Bay remained non-existent or extremely rare until 2014^15^. Partly in response to these events, and out of concern for public safety at beaches, in 2018 the California State Senate passed funding measures (SB 840) to expand population monitoring of white sharks at popular recreation areas^29^. In addition to such traditional and official wildlife monitoring programs, community science initiatives are becoming a viable source of in situ data that can document and reflect changes in realized species distributions^30,31^. As nearshore monitoring programs have only recently been implemented, incorporating community science data streams can improve our emerging understanding of the ecological relationship between climate and juvenile white sharks in the Northeast Pacific.

In this study, we integrate community science initiatives, decadal biologging efforts, and mesoscale environmental data to provide a detailed assessment of the thermal preferences of juvenile white sharks in the Northeastern Pacific. We build a thermal suitability model and use this to calibrate the cold edge of their thermal range and track its position as well as their total available thermal habitat area from 1980 to 2019. We hope that this helps to both describe and explain the documented range shift of juvenile white sharks since the 2014–2016 marine heatwave and provides a useful road map for the synthesis of multiple data streams that should advance the use of marine megafauna as sentinels of climate change.

## Results

Though subadult (> 3.0 m TBL) and adult (> 3.8 m for males, > 4.5 m TBL for females) white sharks are common residents of central California and Monterey Bay^7,14,32^, juveniles have been increasingly detected (Fig. 1) since the 2014–2016 marine heatwave^16^. Photogrammetry validated observations and modeled observer records indicate that juveniles < 2.5 m TBL comprise 92.3% and 64.7% of the observed white sharks, respectively, at the primary aggregation site (Fig. 1b). An ensemble index (Fig. 1c) indicates juvenile white shark abundance increased from 2014 to 2019, concentrated in northern Monterey Bay (Fig. 1d). These data combine observer records from recreational fishermen and a community science project, highlighting the value of public engagement in wildlife monitoring and coastal management.

**Figure 2:**
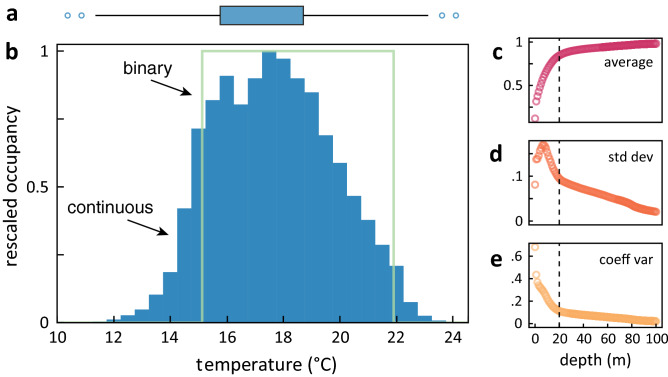
Derived thermal suitability of juvenile white sharks from time-depth recorders. All data are derived from onboard sensors from electronic tags on 14 juvenile white sharks deployed from 2001 to 2019 in southern and central California (Table S1, Figure S1). **a** Summary boxplot where the blue box is the core 50% interval, whiskers are the 95% interval, and hollow circles are extreme temperature values. **b** Binary (green line) and continuous model (blue histogram) of thermal occupancy. Binary model is the realized thermal limit based on the occupancy-weighted 95% interval (15.1–21.9° C). The continuous model reports thermal occupancy continuously, rescaled from the time-at-temperature histogram (Figure S4), here binned every 0.5° C. **a,b** were calculated from temperatures sensed at 0–20 m depths, a criterion determined from the natural breaks in the **c** average, **d** standard deviation and **e** coefficient of variation of time-at-depth between individual sharks.

More than 22 million continuous temperature and depth records from 14 electronic tags reveal the temperature preferences of juvenile white sharks (Fig. 2). Simple boxplots (Fig. 2a) and binary models (Fig. 2b) of thermal preferences provide succinct data summaries (min = 10.5 °C, max = 24.7 °C, median = 16.7 °C, 95% interval = 15.1–21.9 °C). Comparing the mean, SD and CV of the cumulative time spent at ≤ *n* depths between tagged sharks reveals a common break at 20 m and suggests that records from 0 to 20 m broadly constitute near-surface behavior (Fig. 2c-e). A continuous thermal suitability model from these pooled (n = 14 sharks) records at 0–20 m depth provides a detailed thermal habitat for juvenile white sharks consistent with previous independent studies^13,15^ in the Northeast Pacific population (Fig. 2b).

**Figure 3:**
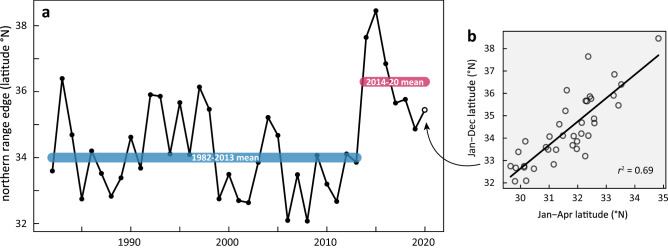
The cold range edge migrates poleward during the 2014–2016 North Pacific heatwave. **a** Annual mean position (filled circles) of the daily center of gravity for the cold edge of the binary thermal suitability model (Fig. 2b) over 1982–2020. The 1982–2013 mean position (blue line) at 34.0° N is near Point Conception (34.4° N), and consistent with historical records that juvenile white sharks largely remaining below this landmark. The 2014–2020 mean position (red line) at 36.4° N is consistent with juvenile white sharks being common in northern Monterey Bay (36.9°N) during that time. **b** Full-year forecast for 2020 (hollow circle in **a**) was calculated from the available Jan-Apr data, and the linear relationship (r^2^ = 0.69, p < 0.001) between this value and the full year using 1982–2019 records.

Cold range edges have been shown to closely track how marine fishes respond to climate change^33^. The center of gravity of the cold edge (*t*_*lat*_) of the continuous thermal suitability model varied considerably from 1982 to 2019. This value had its lowest position in 2008 at 32.0° N, and most northerly position in 2015 at 38.5° N (Fig. 3a). From 1982 to 2013, our models indicate that the mean cold edge position was 34.0° N. This is remarkably consistent with historical population accounts^32^ that juvenile white sharks largely remained south of near Point Conception (34.4° N)^13,14^. Point Conception is a terrestrial headland and is considered a marine biogeographic boundary that sharply separates the warmer waters of the southern California bight, from the northern remnant of the California Current Ecosystem. From 2014 to 2020, the mean position moves 240 km north of Point Conception to 36.3° N. Regression analyses revealed that the Jan-Apr *t*_*lat*_ played a statistically significant role in determining the Jan-Dec *t*_*lat*_ (mean Jan-Apr/Jan-Dec ratio = 0.95, *r*^2^ = 0.69, *p* < 0.001; Fig. 3b), allowing us to forecast *t*_*lat*_ in 2020 at 35.34°N. This poleward thermal habitat shift accompanies the 2014–2016 marine heatwave^16^ and is consistent with the emergence of juvenile white shark observations in northern Monterey Bay (Fig. 1).

**Figure 4:**
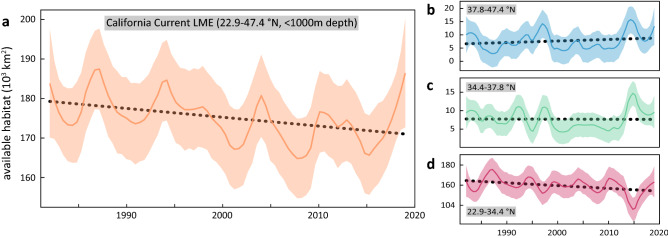
Change in availability of the North Pacific juvenile white shark thermal habitat from 1982–2019. Time series of annal spatial extent of juvenile thermal habitat in **a** the entire extent of the California Current Large Marine Ecosystem (LME) and separately calculated **b–d** in three distinct latitude regions, excluding depths > 1000 m. **a** Across the entire LME the linear change is − 223.2 km^2^ year^−1^. **b** In the northern region (San Francisco to the LME northern boundary) the linear change is + 58.3 km^2^ year^−1^, **c** in central California (Point Conception to San Francisco) there is negligible change (− 3 km^2^ year^−1^), and the historical juvenile white shark range (southern LME boundary to Point Conception) contracts − 275.2 km^2^ year^−1^. While the 2014–2016 North Pacific marine heatwave shifted the thermal habitat poleward (Fig. 3) total available habitat during this time was nearly the lowest recorded. Latitudinal break points in panels a–d represents north–south extent of California Current LME, San Francisco to the California LME northern boundary, Point Conception to San Francisco, and southern California Current LME boundary to Point Conception.

Though the juvenile white shark thermal habitat migrated poleward from 1982 to 2019, the overall available habitat declined. Consistent with previous tagging studies^12,14,15^, regions with the highest thermal suitability were located in the Southern California Bight (“SCB”) and northern Baja California (Figure S6). During our study, the median annual thermal habitat area in the Northeast Pacific was 175,138 km^2^, following a quasi-decadal oscillation, and contracting 223 km^2^ annually (Fig. 4a). The lowest daily habitat available we observed occurred on 15 September 2015 (59,030 km^2^, Figure S6) during the peak of the heat wave event, when the 5-day SST anomaly reached a maximum of + 6.2 °C^19^. In our 38-year data series, the 7 days with the smallest recorded available habitat occur in the last 5 years, from 2015 to 2019 (Table S2). Temporal trends in the historical (− 275.2 km^2^ year^−1^), emergent (− 2 km^2^ year^−1^), and future (+ 58.3 km^2^ year^−1^) habitat regions of juvenile white sharks vary (Fig. 4b-d). Together, however, the net effect of these changes is the poleward expansion and spatial contraction of the thermal habitat consistent with warming (Figure S7).

## Discussion

Climate change is redistributing marine species and ecosystems, completely altering the present and future outlook of commercial and protected species^6,20,34^. Understanding how particular species are affected by climatic changes, however, requires first deriving their habitat and association with environmental conditions empirically through observations^35,36^. Our objective in this study was first to generate a local index of abundance that may describe the recent emergence of juvenile white sharks in Monterey Bay (Fig. 1) and second to examine whether this might be consistent with movements of their thermal habitat cold edge (Fig. 3). The Monterey Bay ecosystem is roughly 2.5° north (~ 600 km linear coastline or ~ 280 km straight line) of what historically has been considered beyond the northerly range limit of juvenile white sharks < 2.5 m TBL in the Northeast Pacific population^13–15^. The increasing presence of this demographic since 2014 in this novel region therefore represents a major geographic shift that carries significant ecosystem consequences. This is further important as juvenile sharks typically remain spatially segregated from adults in geographically distinct nursery areas^12,13^. However, future climate projections of the California Current system^6^ suggest this shift and contraction might be an enduring pattern that is here to stay. Future studies that incorporate population dynamics^37^ will be helpful in further elucidating the long term impacts of N Pacific warming to the changing habitat suitability of the California Current LME for juvenile white sharks.

White sharks appear to exhibit an ontogenetic shift at the age of 3–4 years, at 2.5–3 m TBL, resulting in habitat as well as dietary changes^14^. Piscivorous juveniles were historically restricted to the warm coastal waters in the far southern region of the California Current system (22–34° N; see Figure S5). Juvenile and subadult white sharks typically transition to the colder California waters to the north where they seek larger, energy-rich prey including pinnipeds and stranded cetaceans^14,38,39^. The northward expansion of the thermal habitat for piscivorous juvenile white sharks therefore can alter the established predator–prey relationship along California coastal environments, potentially introducing a new source of natural mortality to commercial forage fish and threatened salmonid populations in central California^40^. Beyond novel impacts to fish populations and fisheries, there may also be implications for protected species conservation and nearshore ecosystem dynamics. The chronology of the poleward shift in juvenile white sharks that we describe here corresponds with, and may help explain, a significant increase in Southern sea otter (*Enhydra lutris nereis*) mortality from white shark bites^39^.

As the Southern sea otter population today is distributed from 34 to 37° N^41^, sea otters have not substantially overlapped with juvenile white sharks for a century or longer^42^. However, the extensive record of sea otter strandings with shark bite wounds today represents the single greatest mortality source facing the threatened species^41,43,44^. This is significant as it poses a risk to the ecosystem benefits that sea otters provide to nearshore ecosystems^45,46^ as well as to sea otter conservation programs that are actively working to recover otter populations and restore their ecosystem functions^47,48^. Since 2010, shark related otter strandings have been on the rise and initially the seasonal pattern of their occurrence overlapped with, and implicated, adult white sharks arriving in Central California for their annual fall foraging migration^39,49^. Since 2012, however, sea otters are being bitten by white sharks throughout the calendar year^39^, including the spring and summer months when adults white sharks are largely absent from the Central California coast^49^. At the same time, the significant loss of *Macrocystis* and *Nereocystis* kelp forest canopies in California has removed sheltering habitats and exposed otters to ambush predators like white sharks^41^.

While these factors working together might explain the increase in shark bites to otters throughout their range, it does not explain why juvenile white sharks, including YOYs, are now in Monterey Bay (Fig. 1). To support this, we note two additional facts. First, while white sharks bite sea otters, these encounters are non-consumptive, presumed investigative, and do not have any function as forage since otters insulate with fur rather than blubber^39^. Therefore, sea otters are not a food source for any life stage of white sharks and present no mechanism to explain juvenile white shark range expansion. The second is that while California sea otter population grew substantially from 1989 to 2014 and expanded their geographic range, the sea otter population density in northern Monterey Bay (where we documented the recent increase in juvenile white sharks) was essentially unchanged during this period^41^. Rather, our tag-derived calculation of the cold edge of the juvenile white shark thermal habitat, and its poleward shift since the 2014–2016 heatwave provide a more compelling explanation of the poleward shift in these endotherms at a younger age than previously recorded.

The growing presence of juvenile sharks above 34° N suggests that climate change may be revising basic aspects of the established spatial population structure for this white shark population, and perhaps others. The occurrence of YOY sharks in Monterey Bay after 2014 (Fig. 1b-c) may reflect local pupping or YOYs traveling from a distant southern nursery area. While we cannot resolve this question in the present study, it is likely that Monterey YOYs immigrated from an emergent white shark nursery in the Southern California Bight, 600 km to the south. Preliminary acoustic records further indicate YOYs have migrated to Monterey Bay (O. Sosa pers. comm.) from the Bahia Sebastian Vizcaino nursery area in Mexico^12^ (28.6° N, 114.84° W) 1,300 km south of Monterey Bay. Though juvenile white sharks < 2.5 m TBL have a clearly defined thermal affinity (Fig. 2), the role of recurring seasonal migrations and behavior is unresolved. In addition, though our mesoscale model output (Fig. 3) successfully predicts the timeline of juvenile white sharks in Monterey Bay, these sharks may not continuously occupy the entire corridor of their thermal habitat. Rather, juvenile white sharks appear to be congregating in a single location in northern Monterey Bay (Fig. 1d), a sheltered bight typified by exceptionally warm water temperatures^50^. Targeted in-water monitoring combined with electronic tagging and fine scale environmental modeling should help resolve such questions.

Our results also indicate an overall compression of thermal habitat for juvenile white sharks from 1982 to 2019, particularly in the southern portion of the range (Fig. 4a,d). During this period, there is an apparent growth of habitat at the northern edge of the California Current system and dominant reduction of suitable habitat in the south (Fig. 4b-d). This growth captures a region of suitable habitat that has been isolated by a 1,000 km expanse of inhospitably cool water (Figure S5) and is therefore disconnected and inaccessible to juveniles from the southern range. While the 2014–2016 North Pacific marine heatwave resulted in the smallest juvenile habitat area that we observed (Fig. 3), there may be cascading effects beyond white sharks. Finer scale analyses, for example, show that such extreme events compress multiple populations in high densities into confined regions, impacting commercial fisheries and protected species^51^.

While we have demonstrated that juvenile white sharks have experienced a dramatic range shift in recent years, corresponding to and seemingly the result of the rapid warming of the California Current, a remaining question is whether the proposed patterns are the result of human-caused global warming. Anthropogenic climate change is considered the primary driver of rapidly increasing upper ocean heat content^52^. This increased heat correspondingly impacts global ecosystems through changes in both mean climatic conditions and climatic variability^3^. however, species typically respond more strongly to extremes than gradual changes in mean conditions^53^. In reality, both are occurring simultaneously, there is both a synoptic increase in mean temperatures coinciding with anomalously warm and extreme events^2,54,55^. Marine heatwaves like the 2014–2016 event in the North Pacific are prolonged, more frequent, and made more intense with anthropogenic climate change^55^.

The emergence of juvenile white sharks in Monterey Bay was unexpected, sudden, and outpaced established scientific monitoring programs. As a result, we developed an index of abundance from community science and recreational fishery records and our project highlights the strategic importance of such initiatives. While many shark populations are threatened and data poor^56^, dedicated fishery-independent monitoring programs face many logistical challenges^57^. Therefore, there is a critical need to incorporate innovative and open-access alternatives to supplement population monitoring, especially at spatial scales relevant for particular management concerns^58,59^. In this vein, community-based biodiversity monitoring may provide an efficient and nimble alternative to more conventional wildlife monitoring programs. For marine megafauna, community science programs have helped document range shifts due to climate change aiding wildlife life managers with important, low cost information about species and their movements^39,41,60,61^. Since many community science records are often opportunistic and lack standardized sampling protocols^62,63^; however, they frequently require post-hoc treatments (see Methods) for quality assurance, formal scientific analysis and management interpretation. Nonetheless, such data streams may have great utility in filling data gaps, providing novel insights and perhaps helping prioritize research infrastructures and resources where there are emerging needs. Beyond providing data, community science initiatives hold additional value as they help to generate public engagement that can increase stakeholders and support for conservation^30^.

## Methods

### White shark observations

We compiled white shark abundance and length observations from field surveys and by curating a community science project. From 2009 to 2019, one of us (EM) kept detailed logbook records of white shark observations during 198 recreational trips in Monterey Bay. With minor variations, all trips used the same vessel (Davis Cortez, Radoncraft, 22′), began and ended in the same port (Moss Landing harbor, 36.80° N, 121.78° W) and followed a similar transect path to a shipwreck reef (SS Palo Alto, 36.96° N, 121.91° W) during daylight hours. From 2009 to 2014, as sharks were uncommon, 1–2 observers manually counted and photographed sharks as they basked near the surface. From 2015 to 2019, observers used a boat-based aerial drone (DJI Phantom4 Pro, 20Mp camera, 4 K video) operating at < 120 m altitude (FAA# FA3T3FRLF7) to photograph and count sharks and estimate body lengths near the shipwreck reef. Logbooks recorded the total number of sharks sighted per trip, and when available listed minimum (*min*) and maximum (*max*) lengths for outliers. As a standard metric of shark abundance, we calculated from these records the total number of white sharks observed per trip.

To understand size composition, we developed a bootstrapping procedure to expand the sparse logbook records using random variates to account for measurement error. For each shark length measurement, this routine generated 1000 random variates from a normal distribution with parameters *μ* and *σ*. Given our (EM) anecdotal observations that 80% of the sharks during the study measured 2.1–2.7 m, we ran 800 of the simulations with *μ* = 2.3 and *σ* = 1, and the remaining simulations with *σ* = 1 and where *μ* was equally split between *min* and *max* recorded lengths. On 9 trips in 2018–2019, we validated shark length estimates with photogrammetry, using drone surveys to capture reference lengths placed alongside surface basking sharks. We plotted the modeled length density (*n* = 403 sharks) against the photogrammetry-validated length density (*n* = 65 sharks), comparing both to accepted length-stage categories for Northeast Pacific white sharks^12,15^.

As an independent measure of juvenile white shark abundance in Monterey Bay, we sought public observations through a community science project. Using the iNaturalist platform^64^, we individually curated user-submitted, geo-referenced images that had previously been taxonomically verified by the iNaturalist user community. From a total of 72 contributed observations excluded subadult and adult (> 3 m TBL) demographics, as well as sharks occurring north of Monterey Bay. This yielded 53 quality-checked and vetted observations from 2012 to 2019 (available at: https://bit.ly/2Px15zo). We report abundances as observations year^−1^, rescale both these and the logbook abundance data from 0 to 1, and fit a single loess model^61^ to both data series as an ensemble index of white sharks in Monterey Bay.

### Deriving thermal suitability models

A two decade biologging program provided a robust data series of the three-dimensional movements and the thermal affinity of juveniles (including neonates, YOY, and small juveniles; 1.4–2.0 m TBL; see Table S1) white sharks in California^14,15,60^. Juvenile white sharks experience a wide temperature range (6–26 °C) throughout the water column while occupying a more specific band (16–20 °C) of preferred temperatures^15^. We quantified observed species occupancy-temperature relationships using both binomial and continuous thermal suitability models. We used temperature data from pop-up archival tags (PAT, Wildlife Computers) deployed on 14 white sharks deployed from 2001 to 2015 (see Table S1, Figures S2 & S3)^15,60^. Deployments originated in the Southern California Bight (SCB), but sharks subsequently ventured throughout their historical range, broadly reflecting the thermal preference of this demographic in the Northeast Pacific.

To capture accurate behaviors, we applied a number of routines to the raw tag data. First, we excluded all tag records both before tag deployment and after tag detachment. Next we applied a correction to the depth series to account for drift in the mechanical pressure sensor following previous studies^65^. Building on previous approaches^15^, we determined juvenile surface temperature preferences by analyzing time spent over the tag-derived temperature gradient. We derived the vertical window considered to encompass surface behavior from a combination of oceanography and behavior. In the California Current system, the mixed layer depth is generally below 50 m, above which temperatures are well mixed^21^. We calculated the cumulative time each individual shark spent at depths ≤ *n* m, where *n* ranged from 0 to 287 m, the observed extremes. To account for individual variability, we then computed the mean, standard deviation (SD), and coefficient of variation (CV) among individuals for each depth and compared the form of these curves across depths. Natural breaks in the mean, SD and CV curves determined the depths considered to include near-surface behavior.

We used the empirical temperature records observed at these tag depths to generate a thermal suitability. Tag deployments were seasonally variable and duration less than one year (mean ± SD: 117.5 ± 80 days). To avoid individual and seasonal biases, we equally weighted observations from each month and each tag, aggregating the observations (*n* = 2.28 × 10^7^) in 0.5° C bins over the recorded temperature gradient (10.6–24.7 °C, see Figure S4). The binary thermal suitability model is the upper and lower thermal limits based on the occupancy-weighted 95% interval, calculated using the R *reldist* package^66^. The 2.5 quantile from this model determined the cold edge of the juvenile white shark thermal habitat. The continuous thermal suitability model provides a continuous thermal habitat suitability (from 0 to 1), yielding more discriminating and precise preferences across all temperatures.

### Determining range limits and habitat availability

Having derived a thermal suitability for juvenile white sharks, we compiled SST observations in the Northeast Pacific from 1982 to 2019 to assess thermal habitat availability (Figure S5). NOAA’S daily Optimum Interpolation Sea Surface Temperature (OISST.v2, 0.25°) integrates SST records from multiple (satellite, vessel, buoy) platforms providing historical environmental data from September 1981 to April 2020^67,68^. Following previous approaches^15,69^, we trimmed these SST data to 0–1000 m depths to avoid nearshore masking of environmental data products using GEBCO bathymetry data^70^.

We tracked the northern range limit (*t*_*lat*_) for juvenile white sharks by calculating the latitudinal center of gravity^71^ of the cold edge of the realized thermal niche through time, using the formula:

$${t}_{lat}= \frac{\sum_{i=1}^{k}\left({lat}_{i}\times {t}_{i}\right)}{\sum_{i=1}^{k}{t}_{i}}$$

where *lat*_*i*_ are for OISST.v2 grid *i*; *t*_*i*_ denotes a calculated binary cold-edge index (0 or 1) at OISST.v2 grid *i*, and *k* is the total number of OISST.v2 cells (n = 657) in the raster grid. As 2020 records were incomplete at the time of this analysis, we extrapolated its value (*t*_*lat_2020*_) based on the partial (Jan-Apr) to full (Jan-Dec) year relationship of *t*_*lat*_ from 1982 to 2019. We average *t*_*lat_2020*_ from two methods—the mean annual value of the Jan-Apr *t*_*lat*_ / Jan-Dec *t*_*lat*_ quotient and a simple linear regression.

We combined the continuous thermal suitability model and the OISST.v2 data to calculate the availability of juvenile white shark habitat over time. For each daily OISST.v2 raster, we converted the SST values in each cell with the model derived thermal suitability. We rescaled the rasters to 0–1 and multiplied the resulting value by cell area (range 522–710 km^2^), summing all raster values. This provides a daily assessment of available habitat that is thermal suitability-weighted according to tag-derived occupancy. We summarized available habitat in three relevant regions of the California Current Large Marine Ecosystem (LME). These areas reflect the historical range (22.9–34.4° N, south LME boundary to Point Conception), the emergent range (34.4–37.8° N, Point Conception to San Francisco), and perhaps the future range (37.8–47.4° N, San Francisco to the north LME boundary). All analyses and visualizations were conducted in the R environment^72^.

## Supplementary Information

Supplementary Information.

## Data Availability

All data used in this study are available at a third-party open access repository (https://osf.io/vcwjp/) and at GitHub (https://bit.ly/3mCzlbF).
