# Supplementary material for: North Pacific warming shifts the juvenile range of a marine apex predator
Source: Sci Rep. 2021 Feb 9;11:3373. doi: 10.1038/s41598-021-82424-9 (PMC7873075; doi:10.1038/s41598-021-82424-9)
Supplement: Supplementary file 1 — Supplementary Information. [file 41598_2021_82424_MOESM1_ESM.docx]

**SUPPLEMENTARY ONLINE MATERIALS ACCOMPANYING THE ARTICLE:**

**“North Pacific warming shifts the juvenile range of a marine apex predator”**

**AUTHORS:** Kisei R. Tanaka,^1^* Kyle S. Van Houtan,^1,2^ Eric Mailander,^1^ Beatriz S. Dias,^1^ Carol Galginaitis,^1^ John O’Sullivan,^1^ Christopher G. Lowe,^3^ and Salvador J. Jorgensen^1,†^*

**AFFILIATIONS:** ^1^ Monterey Bay Aquarium, Monterey, California 93940 USA; ^2^ Nicholas School of the Environment, Duke University, Durham, North Carolina 27708 USA; ^3^ Department of Biological Sciences, California State University Long Beach, Long Beach, California 90815 USA.

* Send correspondence to: kisei.tanaka@gmail.com, salvador.jorgensen@gmail.com

^†^ Present address: Institute of Marine Sciences, University of California, Santa Cruz, California 95064 USA

**This document contains;**

- **Supplemental tables 1-2**
- **Supplemental figures 1-5**

**Table S 1. Summary table of recordings from pop-up archival tag (PAT, Wildlife Computers) deployments on white shark *Carcharodon carcharias* in the Northeast Pacific.**

| **ID** | **n** | **Date** | | **Sex** | **Release location** | | **Pop up location** | | **Total_length (m)** | **Temperature (°C)** | | | **Depth (m)** | |
| --- | --- | --- | --- | --- | --- | --- | --- | --- | --- | --- | --- | --- | --- | --- |
|  |  | **Begin** | **End** |  | **Lon** | **Lat** | **Lon** | **Lat** |  | **Median** | **Max** | **Min** | **Median** | **Max** |
| JWS_02_01 | 34202 | 7/3/01 | 7/26/01 | F | -119.4 | 34.3 | -119.2 | 34.1 | 1.47 | 17.3 | 24.7 | 9.7 | 10.0 | 131.0 |
| JWS_07_01 | 1546560 | 6/14/07 | 12/9/07 | M | -118.5 | 33.9 | -119.3 | 34.2 | 1.45 | 16.8 | 23.5 | 9.2 | 8.0 | 174.5 |
| JWS_08_01 | 2540160 | 6/19/08 | 11/12/08 | F | -116.1 | 30.5 | -118.2 | 33.7 | 1.60 | 18.6 | 24.4 | 9.4 | 3.5 | 232.5 |
| JWS_08_02 | 2540160 | 6/21/08 | 11/14/08 | M | -119.5 | 34.2 | -119.7 | 34.4 | 1.35 | 17.5 | 22.1 | 8.8 | 1.0 | 256.0 |
| JWS_08_04 | 2540160 | 6/27/08 | 11/20/08 | F | -118.5 | 33.9 | -118.2 | 33.2 | 1.54 | 17.9 | 22.9 | 9.0 | 7.5 | 245.5 |
| JWS_08_09 | 2540160 | 8/2/08 | 12/26/08 | M | -118.6 | 34.0 | -119.7 | 34.4 | 1.54 | 16.5 | 22.9 | 8.8 | 1.5 | 273.0 |
| JWS_08_11 | 535680 | 9/8/08 | 10/8/08 | F | -120.4 | 34.1 | -119.8 | 34.4 | 1.36 | 16.8 | 20.6 | 9.3 | 8.5 | 249.5 |
| JWS_09_12 | 223872 | 8/15/09 | 8/27/09 | M | -117.5 | 33.2 | -117.4 | 33.2 | 1.41 | 20.1 | 22.9 | 9.3 | 9.0 | 270.5 |
| JWS_09_15 | 2920320 | 9/27/09 | 3/14/10 | M | -117.5 | 33.4 | -117.6 | 33.4 | 2.24 | 15.3 | 22.8 | 8.0 | 3.5 | 364.0 |
| JWS_10_05 | 102426 | 6/15/10 | 6/26/10 | F | -119.3 | 34.2 | -119.3 | 34.2 | 1.38 | 17.4 | 18.9 | 10.5 | 4.5 | 59.5 |
| JWS_10_10 | 535680 | 7/28/10 | 9/27/10 | M | -119.5 | 34.1 | -119.5 | 34.3 | 1.41 | 15.6 | 19.0 | 10.0 | 2.0 | 117.0 |
| JWS_10_19 | 1537920 | 8/20/10 | 11/16/10 | M | -118.2 | 33.7 | -118.6 | 34.0 | 1.62 | 17.8 | 24.3 | 8.5 | 3.0 | 297.0 |
| JWS_13_01 | 3611520 | 8/29/13 | 3/25/14 | F | -118.4 | 33.9 | -118.5 | 34.0 | 1.59 | 15.8 | 22.6 | 9.3 | 12.5 | 199.5 |
| JWS_14_07 | 1549440 | 11/7/14 | 8/2/15 | M | -121.8 | 36.9 | -118.4 | 33.9 | 1.98 | 17.8 | 24.6 | 8.4 | 6.5 | 282.0 |

**Table S 2. 20 days of smallest available thermal habitat for juvenile white shark in the Northeast Pacific. 7 days with the smallest recorded available habitat occurred during 2015-2019. Availability of juvenile white shark thermal habitat was calculated with the continuous thermal niche model (Figure 2b) and NOAA 0.25° daily Optimum Interpolation Sea Surface Temperature (OISST.v2)^1^.**

| **Date** | **Year** | **Area (km^2^)** | **Average SST (deg C)** |
| --- | --- | --- | --- |
| 9/15/15 | 2015 | 59030.3 | 18.7 |
| 9/14/15 | 2015 | 60860.9 | 18.8 |
| 8/8/18 | 2018 | 61899.5 | 18.6 |
| 8/9/18 | 2018 | 62221.9 | 18.9 |
| 8/10/18 | 2018 | 62523.6 | 19.1 |
| 9/13/15 | 2015 | 63069.0 | 18.9 |
| 9/12/15 | 2015 | 63831.7 | 18.9 |
| 9/5/06 | 2006 | 64599.9 | 17.2 |
| 9/4/06 | 2006 | 66439.7 | 16.9 |
| 9/6/06 | 2006 | 67273.4 | 16.8 |
| 8/11/18 | 2018 | 67570.6 | 19.3 |
| 8/13/18 | 2018 | 68077.4 | 19.0 |
| 7/28/06 | 2006 | 68510.0 | 17.8 |
| 9/16/15 | 2015 | 70835.9 | 18.6 |
| 8/12/18 | 2018 | 70872.0 | 19.1 |
| 9/22/12 | 2012 | 71087.9 | 16.8 |
| 9/20/84 | 1984 | 71196.6 | 18.5 |
| 9/23/12 | 2012 | 71236.2 | 16.8 |
| 9/3/06 | 2006 | 71478.6 | 17.1 |
| 7/29/06 | 2006 | 71644.1 | 17.8 |

**Figure S 1. Spatial extent of the study area in the Northeast Pacific. Bathymetry data were derived from ETOPO1 1 Arc-Minute Global Relief Model^2^.**

**Figure S 2. Pop-up locations (left) and the number of recordings (right) associated with pop-up archival tags (PAT, Wildlife Computers) deployed on 14 white sharks between 2001 and 2015.**

**Figure S 3. Time spent at temperature (10.55-24.7 °C) sensed at 0-20 m with pop-up archival tags (PAT, Wildlife Computers) on 14 white sharks deployed between 2001 and 2015.**

**Figure S 4. Time spent at temperature (10.55-24.7 °C) sensed at 0-20 m. All 14 pop-up archival tags (PAT, Wildlife Computers; *n* = 2.28×10^7^) data were equally weighted, combined, and binned at every 0.5 °C.**

**Figure S 5. Spatial summary of sea surface temperature (SST) in the Northeast Pacific. Left: 1982-2019 SST climatology. Right: SST anomalies map for 2014-2019 relative to 1982-2019 climatology. SST data were drawn from NOAA 0.25° daily Optimum Interpolation Sea Surface Temperature (OISST.v2)^3^.**

**Figure S 6. Spatiotemporal dynamics of juvenile white shark thermal suitability index (0: least suitable – 1: most suitable; < 1000 m depth) between 1982-2019 derived from the continuous thermal niche model (Fig. S3) and NOAA OISST v2 0.25° daily sea surface temperature data. Spatial extent of juvenile thermal habitat varied between 2.71*10^5^ km^2^ (2005-3-16) and 5.91*10^4^ km^2^ (2015-9-15).**

**Figure S 7. Time series of annual sea-surface temperature (SST) anomaly averaged over study area (1982-2019, the dotted line corresponds to a linear increase of 0.01 °C year^−1^). SST data were drawn from NOAA 0.25° daily Optimum Interpolation Sea Surface Temperature (OISST.v2)^1,3^.**

**References**

1 Reynolds, R. & Banzon, V. NOAA Optimum Interpolation 1/4 Degree Daily Sea Surface Temperature (OISST) Analysis, Version 2. *NOAA National Centers for Environmental Information. doi* **10**, V5SQ8XB5 (2008).

2 Amante, C. & Eakins, B. W. ETOPO1 arc-minute global relief model: procedures, data sources and analysis. (2009).

3 Banzon, V., Smith, T. M., Chin, T. M., Liu, C. & Hankins, W. A long-term record of blended satellite and in situ sea-surface temperature for climate monitoring, modeling and environmental studies. (2016).
